# Investigating the spatiotemporal associations between meteorological conditions and air pollution in the federal state Baden-Württemberg (Germany)

**DOI:** 10.1038/s41598-024-56513-4

**Published:** 2024-03-12

**Authors:** Leona Hoffmann, Lorenza Gilardi, Marie-Therese Schmitz, Thilo Erbertseder, Michael Bittner, Sabine Wüst, Matthias Schmid, Jörn Rittweger

**Affiliations:** 1 Institute of Aerospace Medicine, German Aerospace Center (DLR), Cologne, Germany; 2 German Remote Sensing Data Center, German Aerospace Center (DLR), Weßling, Germany; 3 Institute of Medical Biometry, Informatics and Epidemiology, University Hospital Bonn, Bonn, Germany; 4 Department of Pediatrics and Adolescent Medicine, University Hospital Cologne, Cologne, Germany

**Keywords:** Air pollution, Environmental stressors, Meteorological data, Cross-correlation, LISA, Environmental sciences, Health care, Environmental sciences, Health care

## Abstract

When analyzing health data in relation to environmental stressors, it is crucial to identify which variables to include in the statistical model to exclude dependencies among the variables. Four meteorological parameters: temperature, ultraviolet radiation, precipitation, and vapor pressure and four outdoor air pollution parameters: ozone ($\text{O}_3$), nitrogen dioxide ($\text{NO}_2$), particulate matter ($PM_{2.5}$, $PM_{10}$) were studied on a daily basis for Baden-Württemberg (Germany). This federal state covers urban and rural compartments including mountainous and river areas. A temporal and spatial analysis of the internal relationships was performed among the variables using (a) cross-correlations, both on the grand ensemble of data as well as within subsets, and (b) the Local Indications of Spatial Association (LISA) method. Meteorological and air pollution variables were strongly correlated within and among themselves in time and space. We found a strong interaction between nitrogen dioxide and ozone, with correlation coefficients varying over time. The coefficients ranged from negative correlations in January (−0.84), April (−0.47), and October (−0.54) to a positive correlation in July (0.45). The cross-correlation plot showed a noticeable change in the correlation direction for $\text{O}_3$ and $\text{NO}_2$. Spatially, $\text{NO}_2$, $PM_{2.5}$, and $PM_{10}$ concentrations were significantly higher in urban than rural regions. For $\text{O}_3$, this effect was reversed. A LISA analysis confirmed distinct hot and cold spots of environmental stressors. This work examined and quantified the spatio-temporal relationship between air pollution and meteorological conditions and recommended which variables to prioritize for future health impact analyses. The results found are in line with the underlying physico-chemical atmospheric processes. It also identified postal code areas with dominant environmental stressors for further studies.

## Introduction

The human-made climate change threatens human health not only through extreme meteorological conditions, but also through polluted air that often accompanies it^1,2^. There are concrete plans for the reduction of air pollutant concentrations with guidelines from the World Health Organization (WHO)^3,4^ and air quality directive of the European Parliament and the Council^5^. However, there is still a need to better understand outdoor air pollution and their internal relations to prevent potential misinterpretation of the outcomes. In the past, numerous studies were conducted on the effect of air pollution on human health^6–10^. All of them confirm that outdoor air pollution harms human health. Air pollution causes acute and chronic health effects and affects various systems, and organs^8^.

Air pollutants are often released in conjunction, such as nitrogen dioxide ($\text{NO}_2$), carbon dioxide (CO_2_) and particulate matter from combustion processes. The dispersion and deposition of air pollutants through meteorological factors is subject to variation given by emission sources, chemical transformations and average atmospheric lifetime. As a consequence there are spatiotemporal covariations between the various pollutants and meteorological variables.

One of the pollutants is particulate matter with a diameter up to 2.5 ${ }\upmu \text{m}$ ($PM_{2.5}$) which is a variable that has been frequently studied in the literature and has often shown to have a strong negative effect on health. Short-term as well as long-term exposure to $PM_{2.5}$ has an impact on mortality and morbidity^6^. The evidence supports the possibility that both $PM_{2.5}$ and $PM_{10}$ (particulate matter with a diameter up to 10 ${ }\upmu \text{m}$) are associated with increased mortality from all causes, cardiovascular disease, respiratory disease, and lung cancer^11,12^. In Europe in 2020, exposure to $PM_{2.5}$ concentrations above the WHO guideline level of 5 $\upmu \text{g/m}^3$ resulted in 275,000 premature deaths^13^. Most individuals residing in Germany inhabit polluted regions^14^. Air pollution negatively impacts virus-transmitting infections such as influenza^15,16^ and COVID-19^17–19^. Irrespective of which specific health outcome is considered, understanding the interdependencies between environmental variables and the selection of environmental stressors is a key aspect in epidemiological analyses. Previous studies that examined the relationship between meteorological and air pollution variables were conducted in Cairo (Egypt)^20^, China^21^, Rome (Italy)^22^ and Stuttgart (Germany)^23^, for example. Studies on the spatiotemporal variability of tropospheric ozone and nitrogen dioxide are available for Athens (Greece)^24^, major cities in India^25^ as well as in form of worldwide reviews^26^.

In a study on the link between influenza and air pollution^15^, a strong correlation was found between some of the environmental stressors considered, which included air pollutants and meteorological variables. Other studies^27,28^ made use of the principal component analysis (PCA) method for dimension reduction^29^. However, PCA combines pollutants to create principal components. Understanding the individual coefficients can be difficult because they lack interpretability^30^. PCA is unsuitable for our analysis as we want to focus on the specific relationships between and among meteorological conditions and air pollution. Therefore, we opted for temporal and spatial analysis techniques that allow for a more conclusive interpretation of the environmental stressors and investigate the internal dependencies.

The aim of this analysis is to better characterize and identify the spatiotemporal relationships among environmental parameters. Our analysis takes into account multiple stressors and their spatial and temporal connections across the entire state of Baden-Württemberg. The scope is to reduce the number of variables needed in the epidemiological analysis and therefore simplify them and avoid biased results caused by correlated factors.

In the past, research has focused either on analyzing specific cities or studying one environmental factor affecting larger regions. This study is valuable because it considers multiple environmental stressors and covers at the same time the cross-sectional region of Baden-Württemberg (BW). Due to the combination of urban and rural areas including mountainous and river regions, Baden-Württemberg is particularly well suited for analyzing the spatial-temporal relationships between air quality and meteorological conditions. In addition, this paper provides decision support for the selection of environmental variables for future analyses. Understanding how different stressors are interconnected can offer valuable insights to aid in future health impact analyses and assist other researchers in related fields.

Through our spatial and temporal analysis, we have identified distinct differences and similarities in terms of spatiotemporal patterns in environmental stressors. Based on these findings, we suggest prioritizing certain variables for further investigation. Additionally, we categorized postal code areas into specific groups based on their environmental stressor pattern, providing a spatial delineation.

## Methods

### Study area

Baden-Württemberg is Germany’s third most populated state, with an area of approximately 36 thousand km^2^ and a population of approximately 11 million. Geographically, the state is located in southwestern Germany and includes urban and rural areas. The state capital Stuttgart is the largest city in BW with about 630 thousand inhabitants and is located in the center of the state. Other large cities are Mannheim (310 thousand inhabitants), Karlsruhe (308 thousand inhabitants), and Freiburg (231 thousand inhabitants). Especially in the south, southwest, and southeast of the state, there are many rural and mountain regions, including the Black Forest, the Lake Constance, the edge of the Alps, and the low mountain range Swabian Alb. Of all 1101 communities of BW, 586 have fewer than 5 thousand inhabitants^31^.

We obtained a shapefile of the postal code areas in BW from the Esri Germany database^32^ in order to handle the postal code areas.

The population density provides information about the number of inhabitants per km^2^ and was calculated from the available data as follows:

1 $$\begin{aligned} \mathrm {population\, density = \frac{number\, of\, inhabitants}{postal\, code\, area\, (km^2)}} \end{aligned}$$

**Figure 1:**
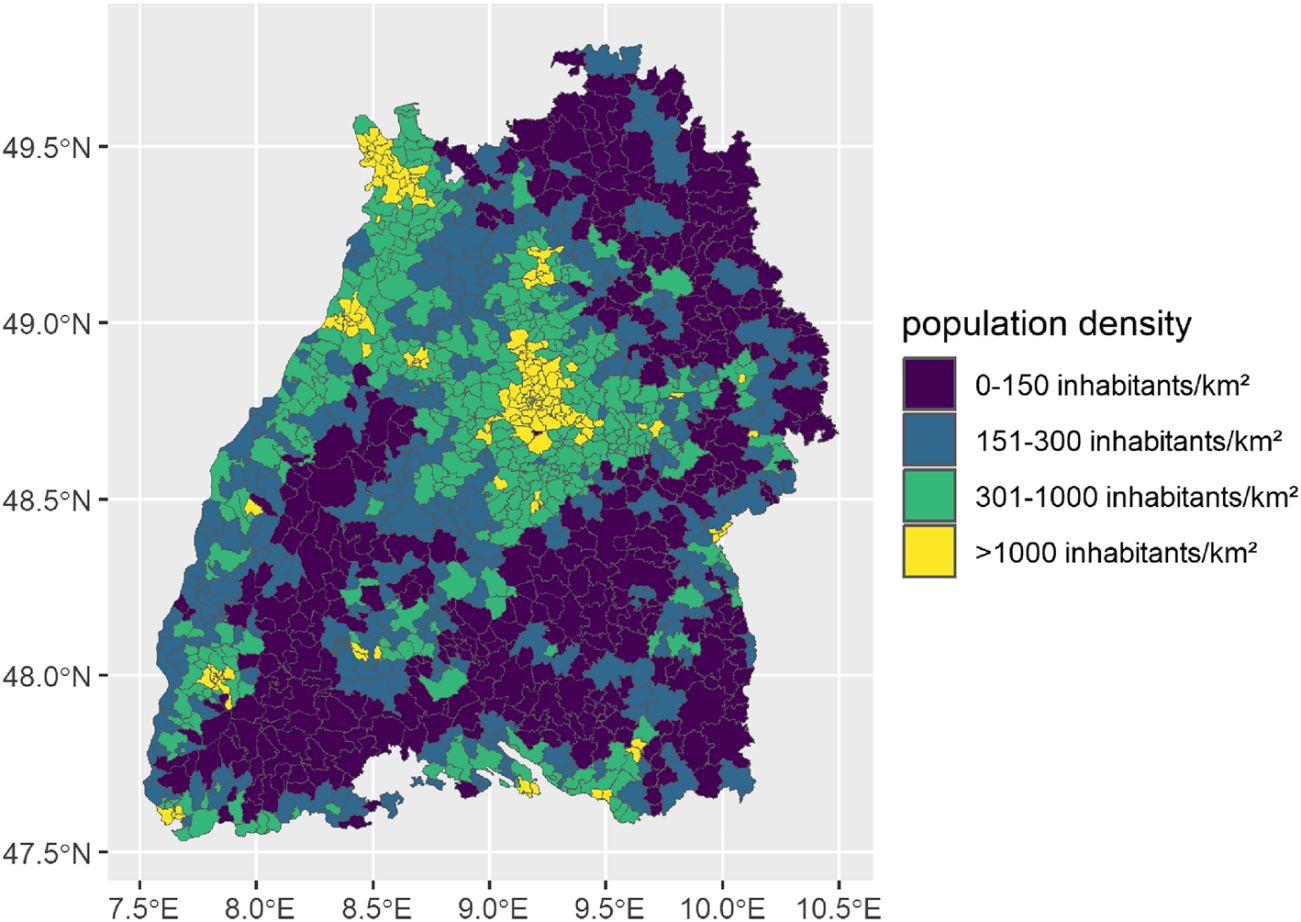
Postal code areas in BW categorized by population density.

Based on a map of population density from the German Federal Institute for Population Research (Bundesinstitut für Bevölkerungsforschung)^33^, we decided to introduce four population density categories. Figure 1 provides a graphical overview of the distribution of the population density categories for BW at postal code level.

Unsurprisingly, postal code areas near cities such as Stuttgart and Mannheim are assigned to category 4, that means densely populated areas. The areas of Black Forest, Swabian Alb, and northeastern of BW predominantly have a population density below 151 inhabitants per km^2^.

### Environmental variables

Eight environmental variables were considered in more detail. These were split into four meteorological parameters [temperature (*Temp*), precipitation (*Prec*), vapor pressure (*VP*) and UV radiation (*UV*)] and four outdoor air pollution parameters [ozone $(\text{O}_3)$, nitrogen dioxide $(\text{NO}_2)$, particulate matter ($PM_{2.5}$, $PM_{10}$)]. Air pollution surface concentrations were retrieved from the European air quality reanalysis dataset provided by the Copernicus Atmosphere Monitoring Service (CAMS)^34^. The data source of the meteorological parameters was the ERA5 reanalysis dataset as provided by the Copernicus Climate Change Service (C3S) of the European Centre for Medium-Range Weather Forecasts (ECMWF)^35^. The datasets had a native spatial resolution of 0.1° × 0.1° and a temporal resolution of one hour. The variables were geographically aggregated to postal code areas as described in^36^. A detailed evaluation of uncertainties of the applied data can be found in^37–39^

**Table 1:** Overview of environmental stressors.

Variable	Parameter	Value
Temp (°C)	Mean (sd)	9.7 (7.7)
	Median [min, max]	9.8 [−18.9, 30.9]
Prec (mm/day)	Mean (sd)	3.4 (6.0)
	Median [min, max]	0.9 [0, 99.3]
VP (hPa)	Mean (sd)	10.0 (4.2)
	Median [min, max]	9.3 [1.0, 24.6]
UV (W)	Mean (sd)	15.4 (9.7)
	Median [min, max]	14.1 [0.4, 37.0]
${\text{O}_3}$ ($\upmu \text{g/m}^3$)	Mean (sd)	51.5 (23.0)
	Median [min, max]	52.4 [0.3, 149.0]
${{\text{NO}_2}}$ ($\upmu \text{g/m}^3$)	Mean (sd)	12.2 (7.3)
	Median [min, max]	10.4 [1.07, 66.4]
${{PM_{2.5}}}$ ($\upmu \text{g/m}^3$)	Mean (sd)	11.0 (6.7)
	Median [min, max]	9.5 [0.7, 72.3]
${{PM_{10}}}$ ($\upmu \text{g/m}^3$)	Mean (sd)	14.9 (8.4)
	Median [min, max]	13.2 [0.9, 83.5]

Overview of meteorological data and air pollutants according to the statistical parameters mean, standard deviation (sd), median, minimum (min) and maximum (max) value. The variables cover all postal code areas in BW and are based on daily measurements from 2010 to 2018.

Table 1 gives an overview of the environmental stressors. Daily mean values between 2010 and 2018 were available for each variable in the table, aggregated at the postal code level in BW and resulting in a total of 3,931,252 data. Next is a brief explanation of the environmental variables^40,41^. The variable *Temp* was measured two meters above the ground. *Prec*, expressed in mm/day, represented the depth of water if it were evenly distributed over the area under consideration. The variable *VP* was a variable constructed from 2*m* dewpoint temperature, as expressed by the following empirical formula^42^:

2 $$\begin{aligned} e=6.112*\text{exp}(\frac{17.67*T_d}{T_d-243.5}) \end{aligned}$$

where e was the vapor pressure in hectopascal (hPa) and $T_d$ was the dew point temperature in °C. For *UV*, the unit was converted from $\text{J/m}^2$ to $\text{W/m}^2$ by dividing the integration time in seconds, resulting in a mean value of 15.4 $\text{W/m}^2$ in the processed data. $\text{O}_3$ is a colorless and toxic gas in the atmosphere close to the ground (troposphere). $\text{O}_3$ is one of the main components of photochemical smog and is produced by complex photochemical processes during intense sunlight. $\text{NO}_2$ is a reactive nitrogen compound that is commonly released from the combustion of fuels in the transportation and industrial sectors. $PM_{2.5}$ and $PM_{10}$ are not single pollutants but a mixture of many components such as sulfates, nitrates, ammonia, sodium chloride, black carbon, mineral dust, and water. Depending on the size of the particles, a distinction is made between $PM_{10}$ and $PM_{2.5}$. Particulate matter is generated, in particular, by combustion processes in motor vehicles, power plants, small combustion plants, domestic heating as well as in metal and steel production.

### Statistical methods

Pearson’s correlation coefficient was used to determine the pairwise correlations between environmental stressors. The strength of correlation is classified as follows: $r > 0.9$ almost perfect, $0.7 < r \le 0.9$ very large, $0.5 < r \le 0.7$ large, $0.3 < r \le 0.5$ moderate, $0.1 < r \le 0.3$ small and $r < 0.1$ trivial^43^. From the pearson correlation coefficient emerges the concept of cross- and autocorrelations. In short, a cross-correlation examines the relationship between two or more parameters over time or in space, whereas an autocorrelation examines the relationship to itself (also possible in time and space). The concept of temporal cross-correlation was used to make more precise statements about the temporal internal dependencies of environmental stressors^22^. In a cross-correlation function (ccf), two time series, *x*(*t*) and *y*(*t*), are examined for correlations with a time offset^44,45^. The formula can be represented as follows:

3 $$\begin{aligned} \textrm{ccf} = \frac{\sum \nolimits _{t=1}^{N-1}[(x(t)- {\bar{x}})*(y(t- \textrm{lag})-{\bar{y}})]}{\sqrt{\sum \nolimits _{t=1}^{N-1}(x(t)- {\bar{x}})^2}\sqrt{\sum \nolimits _{t=1}^{N-1}(y(t- \textrm{lag})-{\bar{y}})^2}} \end{aligned}$$

where $\bar{x}$ and $\bar{y}$ denote the mean over time of the corresponding series, respectively. The time series *x*(*t*) is fixed, and $y(t \pm lag)$ has a time lag, which is possible in both directions, i.e., *x* leads *y* or *x* lags *y*. In a resulting correlation plot, horizontal lines represent the individual correlations of the two time series with the respective time lag.

A local indication of spatial association (LISA) model was used for the spatial analysis. The LISA statistic measures the degree of autocorrelation between a geographical location and its neighbors, identifying so-called hot and cold spots. For instance, hot spots refer to areas with significantly high values that are surrounded by postal code regions with high values. This method was developed by Luc Anselin^46^ and, among others, applied in the context of air pollution^16,47^. Several statistics represent the measure of spatial autocorrelation^48,49^. Here, we used the local Moran statistic, whereby the statistic was applied to each environmental stressor individually. The local Moran’s *I* statistic was given as follows:

4 $$\begin{aligned} I_i = \frac{(x_i-\bar{X})}{S_i^2}\sum \limits _{j=1,j\ne i}^{n} w_{i,j}(x_j-\bar{X}) \end{aligned}$$

where $x_i$ was the stressor concentration at location *i*, $x_j$ was the concentration of spatial lag *j* (neighbors), and $\bar{X}$ was the global mean of the environmental stressor. The spatial weight between *i* and *j* was described by the matrix $w_{i,j}$, and the total number of observations was *n*. $S_i^2$ was a constant for all locations with:

5 $$\begin{aligned} S_i^2 = \frac{\sum \limits _{j=1,j\ne i}^{n}(x_j-\bar{X})^2}{n-1} \end{aligned}$$

Moran’s test uses a null hypothesis of randomly dispersed data. All statistical analyses were conducted using R^50^, and maps and figures 1 to 9 were generated in R version 4.3.0.

## Results

**Figure 2:**
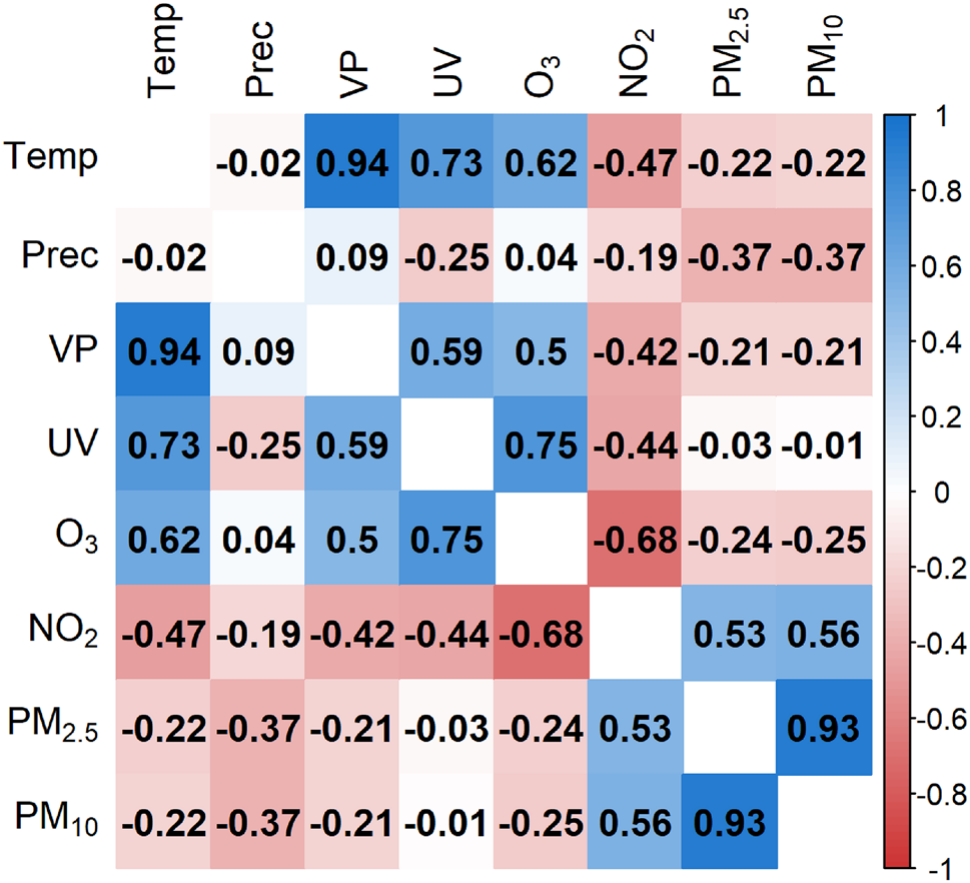
Pearson correlation matrix based on daily measurements from 2010 to 2018 across BW. The more intense the color, the stronger the correlation between the two variables, whereas the color blue indicated positive correlations and red negative correlations.

A matrix of pairwise Pearson’s correlation coefficients between the different environmental parameters was shown in Fig. 2.

The correlations represented associations across the entire observation period, that means over all days from 2010 to 2018 and total BW. As expected the temperature and vapor pressure (correlation coefficient (r) 0.94) and $PM_{2.5}$ and $PM_{10}$ ($r = 0.93$) had a strong positive correlation. In addition, there were other relatively strong correlations. The correlation coefficient between *UV* radiation and $\text{O}_3$ was 0.75. This means that the stronger the radiation was, the higher the $\text{O}_3$ concentration. $\text{O}_3$ and $\text{NO}_2$ are negatively correlated ($r = -0.68$). If one air pollutant was low, the other was high.

### Temporal analysis

**Figure 3:**
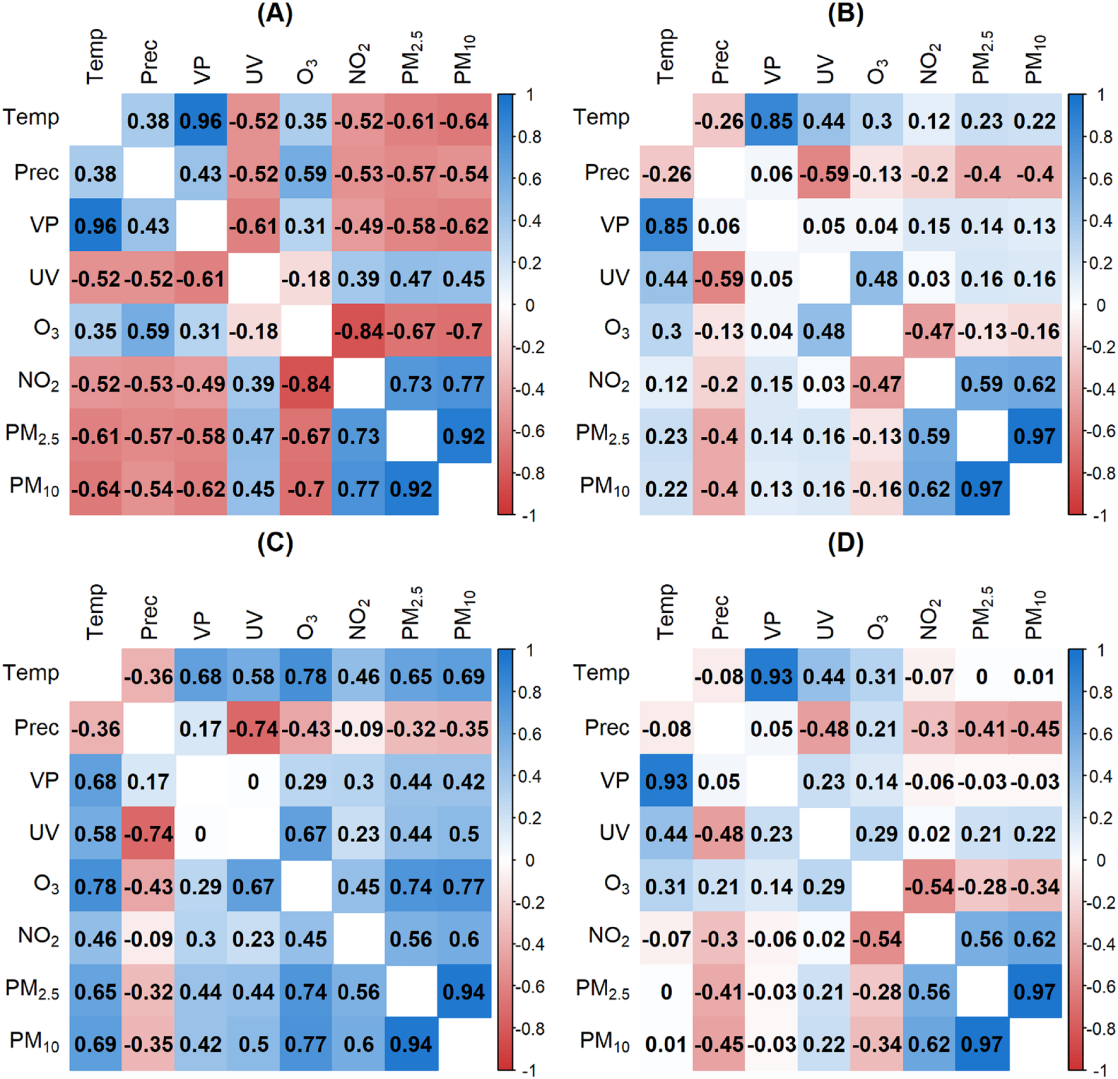
Pearson correlation matrix over months for January (**A**), April (**B**), July (**C**) and October (**D**).

Since this part focuses on temporal relationships, the spatial separation into postal code areas was neglected for this section and the values were averaged over time. It was investigated whether the pairwise Pearson correlations differ across months. We carefully examined correlation plots for all months and decided to include the first correlation plot of each quarter (January, April, July, and October) in the manuscript. Within the manuscript, we do not display all the months explicitly, as some of them represent transitions between the extreme figures shown. The correlation matrices for every month are available in the Supplementary information. The results for the four months January, April, July and October were presented in Fig. 3.

In January, there were many negative correlations. In April, the correlation matrix was primarily characterized by lower correlations. In July, stronger correlations dominate and in October, again, many weaker correlations were visible. The matrices of October and April were overall very similar. They seemed to be intermediate states between the more extreme correlations in January and July.

For $\text{O}_3$, the sign of the correlation coefficient with *Prec* was not constant. The strength of the correlation differed in January ($r = 0.59$), April ($r = -0.13$), July ($r = -0.43$) and October ($r = 0.21$). The medium-large negative correlation between *UV* and *Prec* was almost constant over the months. *UV* and $\text{O}_3$ were slightly correlated in January ($r = -0.18$), moderately positively correlated in April ($r = 0.48$), strongly positively correlated in July ($r = 0.67$), and slightly correlated in October $(r = 0.29$). The variables $\text{O}_3$ and $\text{NO}_2$ were very strongly negatively correlated in January ($r =-0.84$), moderately negatively correlated in April ($r=-0.47$), moderately positively correlated in July ($r=0.45$), and again negatively correlated in October ($r=-0.54$). The correlation between $\text{NO}_2$ and $PM_{2.5}$, as well as $PM_{10}$, was positively correlated over all months. In January, the correlation was stronger, with values around 0.75, than in the other months, with values around 0.6. To summarize, Fig.  3 showed numerous changes of sign over months. Specifically, 14 of the total 28 correlation coefficients showed a change in sign between January and July.

**Figure 4:**
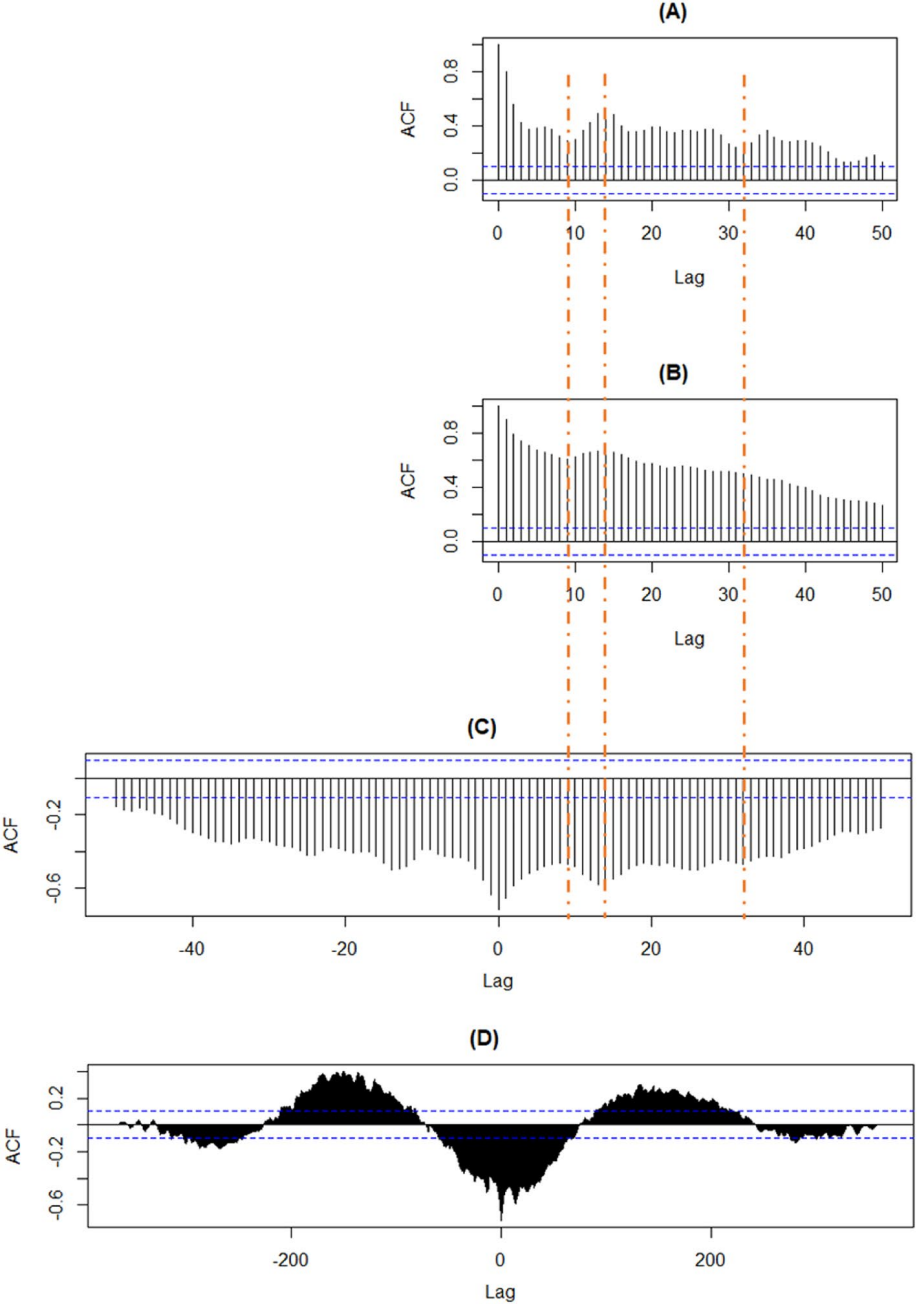
Autocorrelation function for $\text{NO}_2$ (**A**) and $\text{O}_3$ (**B**) of the year 2018. Cross-correlation function (ccf) for $\text{NO}_2$ and $\text{O}_3$ of the year 2018 with 50 days lag (**C**) and 400 days lag (**D**). The time series $\text{O}_3$ was fixed, and the time series $\text{NO}_2$ shifted by lags for the ccf function. Plots (**C**) and (**D**) showed the correlation between $\text{NO}_2$ at time $t\pm lag$ and $\text{O}_3$ at time *t*.

The correlation between $\text{NO}_2$ and $\text{O}_3$ was already prominent in Fig. 3. Therefore, the auto- and cross-correlation relationship between the $\text{O}_3$ and $\text{NO}_2$ variables was depicted in Fig. 4. Overall, the plots by year look very similar, so exemplary, the correlations of the environmental stressors in Fig. 4 were presented for 2018. All graphs had a low point at lag nine and a high point at lag 14. There were noticeable differences in the plots. Specifically, lag 32 in (A) showed a dip that was not present in (B) and (C). In Fig. 4D there was an annual periodicity with the highest positive Pearson correlation coefficient reaching around 0.4 when $\text{NO}_2$ comes before $\text{O}_3$ and approximately 0.3 when $\text{NO}_2$ follows $\text{O}_3$. In addition, there was a pattern that repeats about every 13 days in (C).

**Figure 5:**
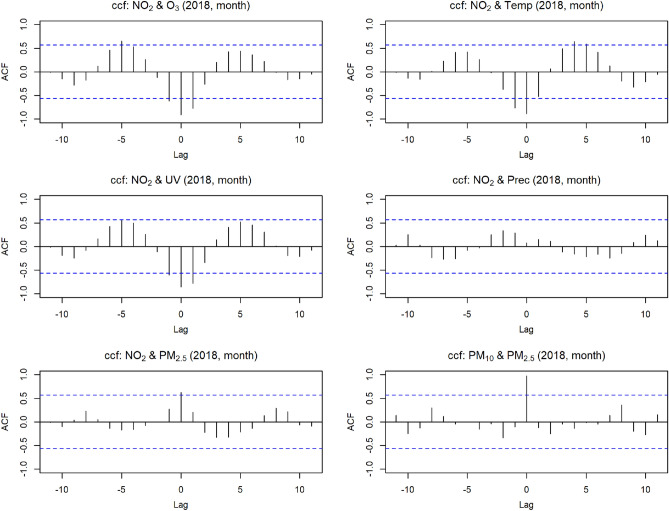
Different cross-correlation functions for the year 2018 (lag = month).

The output of the cross-correlation function between $\text{NO}_2$ and $\text{O}_3$ in Fig. 5 was essentially the same as the day-level correlation in Fig. 4. The figures for $\text{NO}_2$ and *Temp* as well as $\text{NO}_2$ and *UV* looked very similar to $\text{NO}_2$ and $\text{O}_3$. There was also a change in signs and another high point after about half a year in both directions. A small structure in Fig. 5 was seen between $\text{NO}_2$ and *Prec* in the correlation values shifted by months. That means a wave-like structure similar to $\text{NO}_2$ and $\text{O}_3$ can be observed. In contrast to $\text{NO}_2$ and $\text{O}_3$, the correlation coefficient starts in the positive range at lag 0, changes to negative with increasing lags, and returns to positive. However, these were relatively weak correlations. A shift made sense content-wise because rain washes the air clean of pollutants. $\text{NO}_2$ and $PM_{2.5}$ started at lag 0 with a relatively strong correlation value above 0.5. For positive lag values, if $\text{NO}_2$ lags behind $PM_{2.5}$, there was a similar structure to the cross-correlation of $\text{NO}_2$ and *Prec* at low level. The ccf plot for $PM_{10}$ and $PM_{2.5}$ gave a strong correlation at lag 0, followed by no clear structure at the monthly shift.

### Spatial analysis

The aim of the the LISA analysis^46,47^ was to identify the locations of clusters of LISA hot and cold spots of the environmental stressors throughout BW.

**Figure 6:**
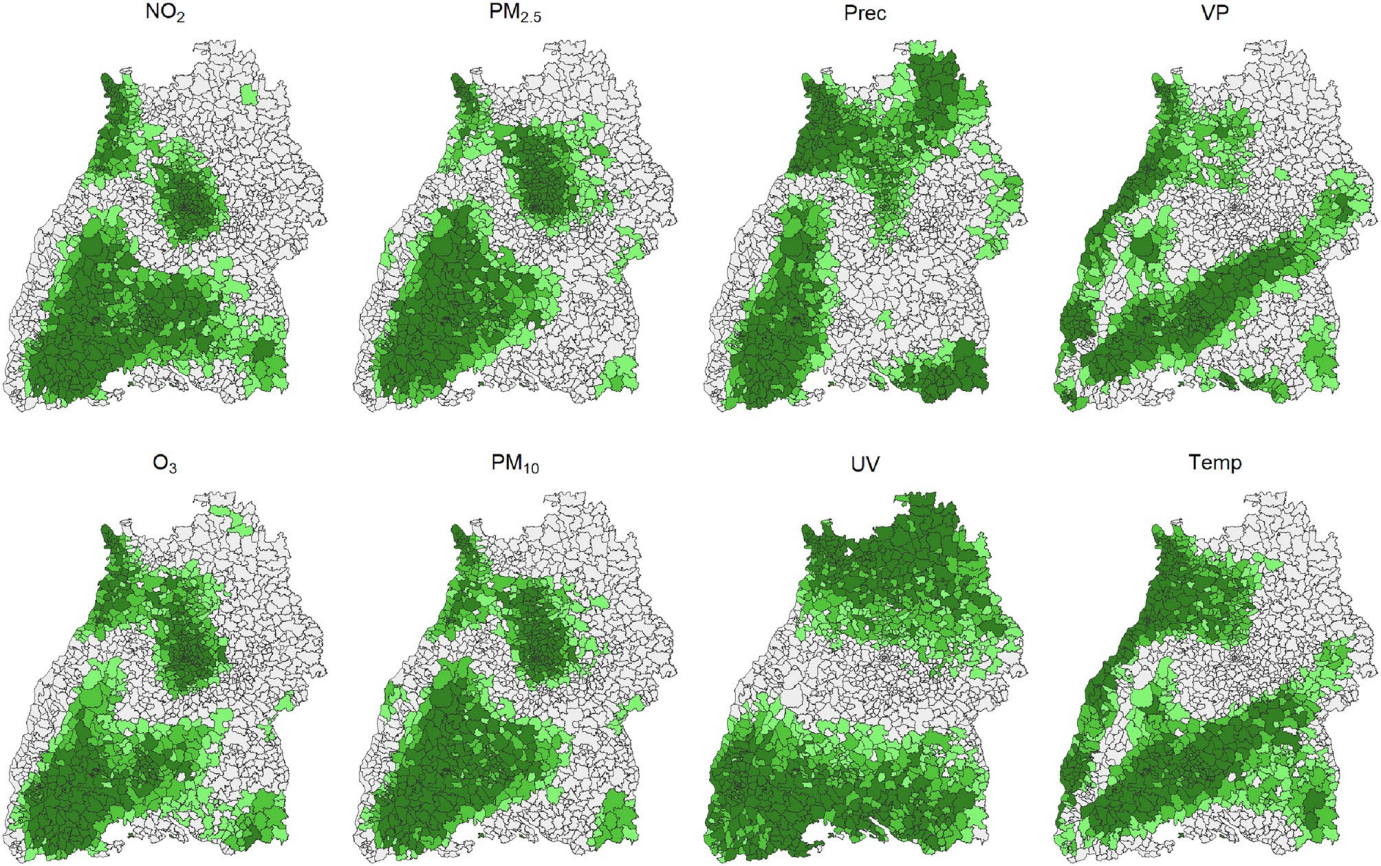
Local significance map of the individual environmental stressors in BW. The figure represented the results of the significance test of LISA analysis using Moran’s I. Different shades of green indicated different thresholds: light green $p < 0.05$; medium green $p < 0.01$ and dark green $p < 0.001$.

**Figure 7:**
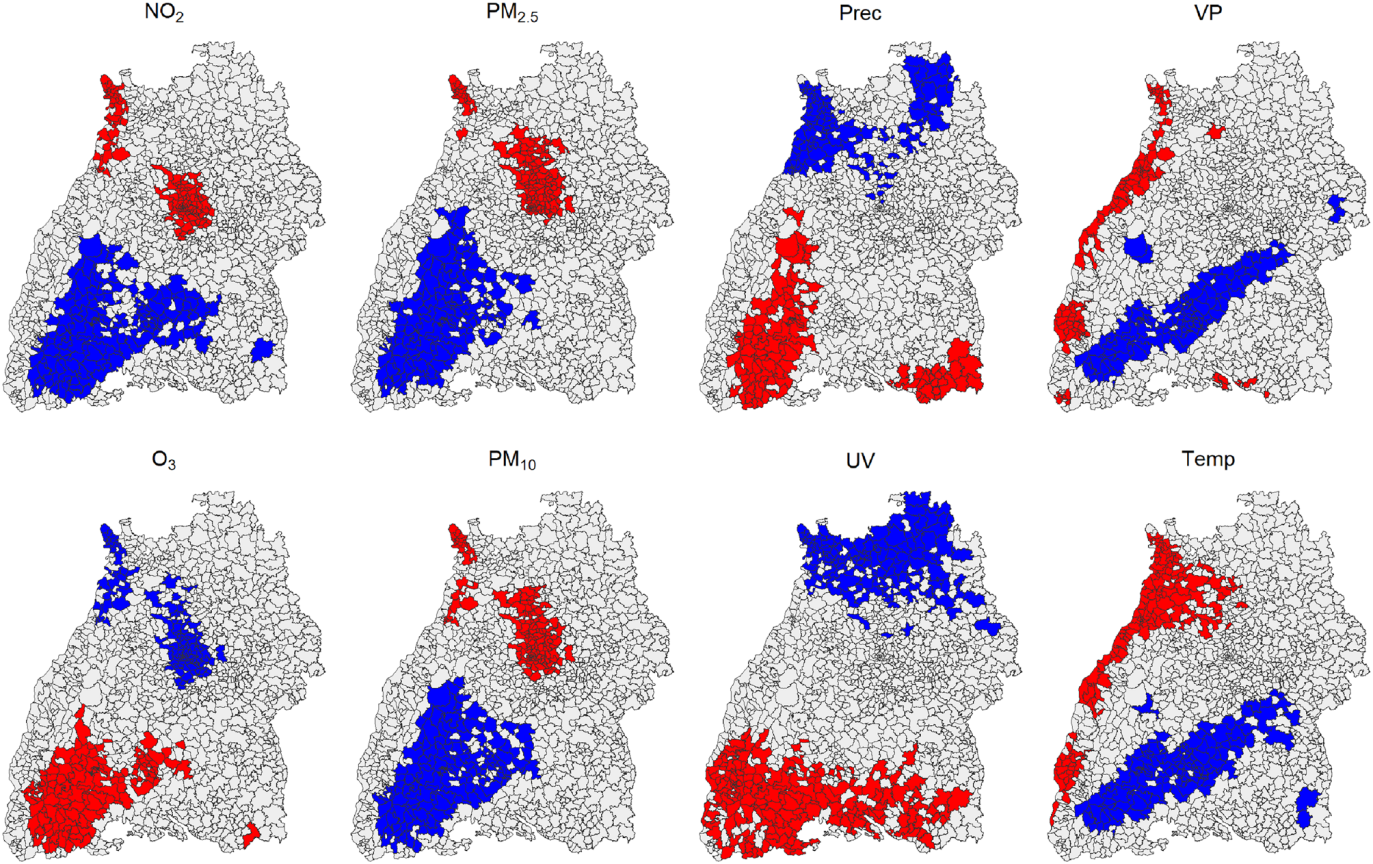
Local Cluster Map of the individual environmental stressors in BW. The significance level was set to $p \le 0.001$. LISA hot spots were red, representing positive spatial autocorrelation with high values. LISA cold spots were blue, representing low values. BW had three postal code areas (78,266, 78,465, 78,479) that were isolated without neighboring postal code areas. We omitted these areas from any further consideration. The estimation was always relative to the mean, as constructed in formula (4). The combination of the red and blue areas results in the dark green areas in Fig. 6.

According to Fig. 6, regions with very high or low population density are most affected by air pollution variables such as $PM_{10}$, $PM_{2.5}$, $\text{NO}_2$ and $\text{O}_3$. The meteorological variables had various patterns. A more detailed representation of the spatial associations was given in Fig. 7 with a significance level set to 0.001. Using local cluster maps, the spatial associations between postal code areas were summarized into LISA hot and cold spots.

As already showed in the local significance map, most air pollution variables showed a similar structure. The following applies to $\text{NO}_2$, $PM_{10}$, and $PM_{2.5}$: In urban areas (Stuttgart + Mannheim region), many postal code areas had neighbors with significantly similar high values. The cold spots geographically contain the Black Forest and parts of the Swabian Alb. For $\text{O}_3$, the distribution of LISA hot and cold spots was similar but reversed. Based on the meteorological variables, different postal code regions stand out, with *VP* and *Temp* showing similar trends. Accordingly, LISA hot spots were located at the western edge of BW. LISA cold spots were found along the Schwäbische Alb. A possible geographical connection could be the differences in altitude of the individual areas. For *UV*, LISA cold spots were located exclusively in the north, and LISA hot spots were exclusively in the south of the state. The Local Moran Map for *Prec* looked similar, i.e., LISA cold spots in the north and LISA hot spots in the south. However, no such clear and extreme assignment as for *UV* was recognizable. This could possibly be explained by the altitude, latitude and local air circulations. Furthermore, areas of the Black Forest showed LISA hot spot postal code areas. One could assume a connection between *Temp* and *UV*. However, this map showed no spatial relationship between LISA hot and cold spots for *UV* and *Temp*.

Pairwise correlations between stressors and cross-correlation plots were examined by the population density. Table 2 showed a summary of environmental stressors split by population density categories.

**Table 2:** Overview of environmental stressors split by population density categories.

Variable	Parameter	1	2	3	4
Temp (°C)	Mean (sd)	9.0 (7.7)	9.7 (7.7)	10.0 (7.7)	10.3 (7.6)
	Median [min, max]	9.2 [−18.5, 29.9]	9.9 [−18.9, 30.8]	10.2 [−18.6, 30.9]	10.4 [−18.2, 30.9]
Prec (mm/day)	Mean (sd)	3.6 (6.3)	3.4 (6.0)	3.3 (5.9)	3.1 (5.6)
	Median [min, max]	1.0 [0, 99.3]	0.9 [0, 98.1]	0,9 [0, 98.0]	0.8 [0, 85.4]
VP (hPa)	Mean (sd)	9.7 (4.2)	10.0 (4.2)	10.1 (4.3)	10.2 (4.3)
	median [min, max]	9.1 [1.0, 24.0]	9.4 [1.1, 24.6]	9.5 [1.1, 24.6]	9.6 [1.1, 24.3]
UV (W)	Mean (sd)	15.5 (9.6)	15.4 (9.7)	15.3 (9.7)	15.2 (9.7)
	Median [min, max]	14.1 [0.4, 37.0]	14.1 [0.4, 37.0]	14.0 [0.4, 37.0]	13.9 [0.4, 37.0]
${\text{O}_3}$ ($\upmu \text{g/m}^3$)	Mean (sd)	54.4 (22.3)	52.0 (22.8)	50.0 (23.2)	47.2 (23.5)
	Median [min, max]	55.1 [0.4, 149]	52.9 [0.6, 148]	51.0 [0.3, 149]	48.3 [0.5, 149]
${\text{NO}_2}$ ($\upmu \text{g/m}^3$)	Mean (sd)	9.6 (5.8)	11.4 (6.6)	13.5 (7.4)	16.5 (7.5)
	Median [min, max]	8.1 [1.7, 31.3]	9.7 [2.0, 38.3]	11.9 [2.5, 45.5]	14.8 [3.3, 53.0]
${PM_{2.5}}$ ($\upmu \text{g/m}^3$)	Mean (sd)	10.5 (6.1)	10.9 (6.3)	11.2 (6.5)	11.6 (8.7)
	Median [min, max]	8.0 [1.1, 66.4]	9.7 [1.1, 60.0]	11.7 [1.1, 66.0]	14.7 [1.4, 66.4]
${PM_{10}}$ ($\upmu \text{g/m}^3$)	Mean (sd)	14.1 (7.9)	14.7 (8.3)	15.3 (8.7)	16.0 (9.1)
	Median [min, max]	12.6 [0.9, 79.6]	13.1 [1.0, 80.0]	13.6 [1.1, 83.5]	14.2 [1.3, 82.5]

Overview of environmental stressors split by population density category 1: 0–150 inhabitants/km^2^, category 2: 151–300 inhabitants/km^2^, category 3: 301–1000 inhabitants/km^2^, category 4: > 1000 inhabitants/km^2^ including information on standard deviation (sd), median, minimum (min) and maximum (max) value. The variables covered all postal code areas in BW and were based on daily data from 2010 to 2018.

$\text{NO}_2$ showed the most remarkable change in concentration across the population density compared to the other environmental stressors with a mean value ranging from 9.6 $\upmu \text{g/m}^3$ in rural to 16.5 $\upmu \text{g/m}^3$ in urban areas. The more urban the area was, the higher the $\text{NO}_2$ concentration. For $PM_{2.5}$ and $PM_{10}$, a similar but less intense increase was observed. $\text{O}_3$ had a reverse effect ranging from 54.4 $\upmu \text{g/m}^3$ to 47.2 $\upmu \text{g/m}^3$. The environmental stressors *UV*, *VP*, *Temp*, and *Prec* were mainly constant over space.

**Figure 8:**
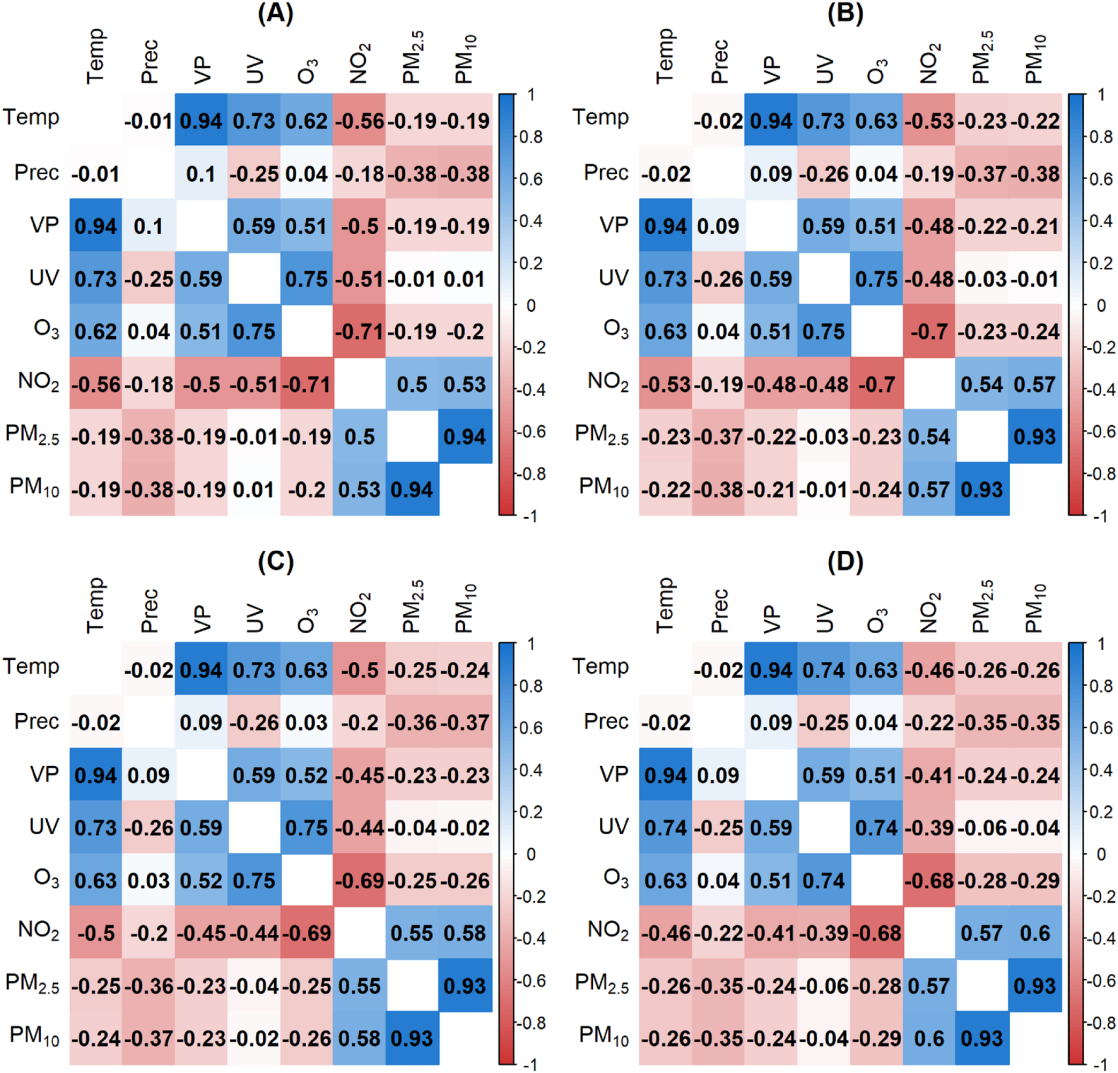
Pearson correlation matrices based on daily data aggregated by population density category 1 (**A**): 0–150 inhabitants/km^2^, category 2 (**B**): 151–300 inhabitants/km^2^, category 3 (**C**): 301–1000 inhabitants/km^2^, category 4 (**D**): > 1000 inhabitants/km^2^.

Figure 8 showed the Pearson correlation matrix for all data separated by population density category. If we specifically compare the most rural and urban areas (categories 1 and 4), we noticed that $\text{NO}_2$ had different correlation values depending on the category. $\text{NO}_2$ correlated more negatively with *Temp* $(r = -0.67)$, *VP* $(r = -0.6)$, and *UV* $(r = -0.5)$ in category 1 of population density than in category 4 with correlation coefficients of −0.55 (*Temp*), −0.48 (*VP*) and −0.45 (*UV*). The correlation between $\text{NO}_2$ and $\text{O}_3$, as well as *Prec*, remained stable mainly across the shifting categories of population density. Between $\text{NO}_2$ and $PM_{2.5}$ as well as $PM_{10}$, the positive correlation increased with rising population density from 0.51 and 0.53 (category 1) to 0.61 and 0.63 (category 4). All other correlations showed only minor variations across population density categories.

The hot and cold spot resulted from the LISA analysis can be used to present a new level of spatial units in addition to the predefined categorical spatial units for population density in Table 2. When comparing the LISA hot and cold spots, it could be seen that they do not always corresponded to categories 1 through 4 of population densities. The parameters *Prec*, *UV*, *VP*, and *Temp*, in particular, differed from the distribution of population density in BW. Additionally, the LISA hot spots do not entirely matched the high population density in Table 2 for the $\text{NO}_2$, $PM_{2.5}$, $PM_{10}$, and $\text{O}_3$ parameters. Table 3 provided an overview of descriptive statistics for the new spatial units as obtained from the LISA analysis.

**Table 3:** Overview of environmental stressors split by LISA spatial units.

Variable	Parameter	LISA hot spots	LISA cold spots	Isolated	Non significant
${\text{NO}_2}$ ($\upmu \text{g/m}^3$)	Mean (sd)	18.5 (8.7)	7.6 (4.6)	11.2 (6.5)	12.0 (6.9)
	Median [min, max]	16.7 [2.5, 66.4]	6.24 [1.1, 42.9]	9.2 [1.7, 40.3]	10.3 [1.1, 66.4]
${\text{O}_3}$ ($\upmu \text{g/m}^3$)	Mean (sd)	59.7 (21.8)	45.1 (23.2)	53.7 (23.5))	51.5 (22.8)
	Median [min, max]	59.5 [1.1, 149]	46.4 [0.5, 136]	55.2 [3.3, 125]	52.4 [0.3, 149]
${PM_{2.5}}$ ($\upmu \text{g/m}^3$)	Mean (sd)	12.0 (7.1)	9.5 (5.9)	11.3 (6.9)	11.0 (6.7)
	Median [min, max]	10.5 [1.2, 63.1]	8.3 [0.8,50.4]	9.8 [1.1, 57.3]	9.6 [0.7, 72.3]

Overview of $\text{NO}_2$, $\text{O}_3$ and $PM_{2.5}$ split by LISA spatial units hot spot, cold spot, isolated and other non significant postal code areas including information on standard deviation (sd), median, minimum (min) and maximum (max) value. The variables covered all postal code areas in BW and included all days from 2010 to 2018. Note that the LISA spatial units differed based on the environmental stressor, as illustrated in Fig. 7. As a result, the quantity of values in each category varied.

We introduced a new level of insight that was defined by the spatial variability of the stressors rather than relying on a predefined quantity such as population density. The strong discrepancy between the mean values was remarkable when looking at the hot and cold spots. These varied from 18.5 to 7.6 for $\text{NO}_2$, 59.7 to 45.1 for $\text{O}_3$, and 12.0 to 9.5 for $PM_{2.5}$.

**Figure 9:**
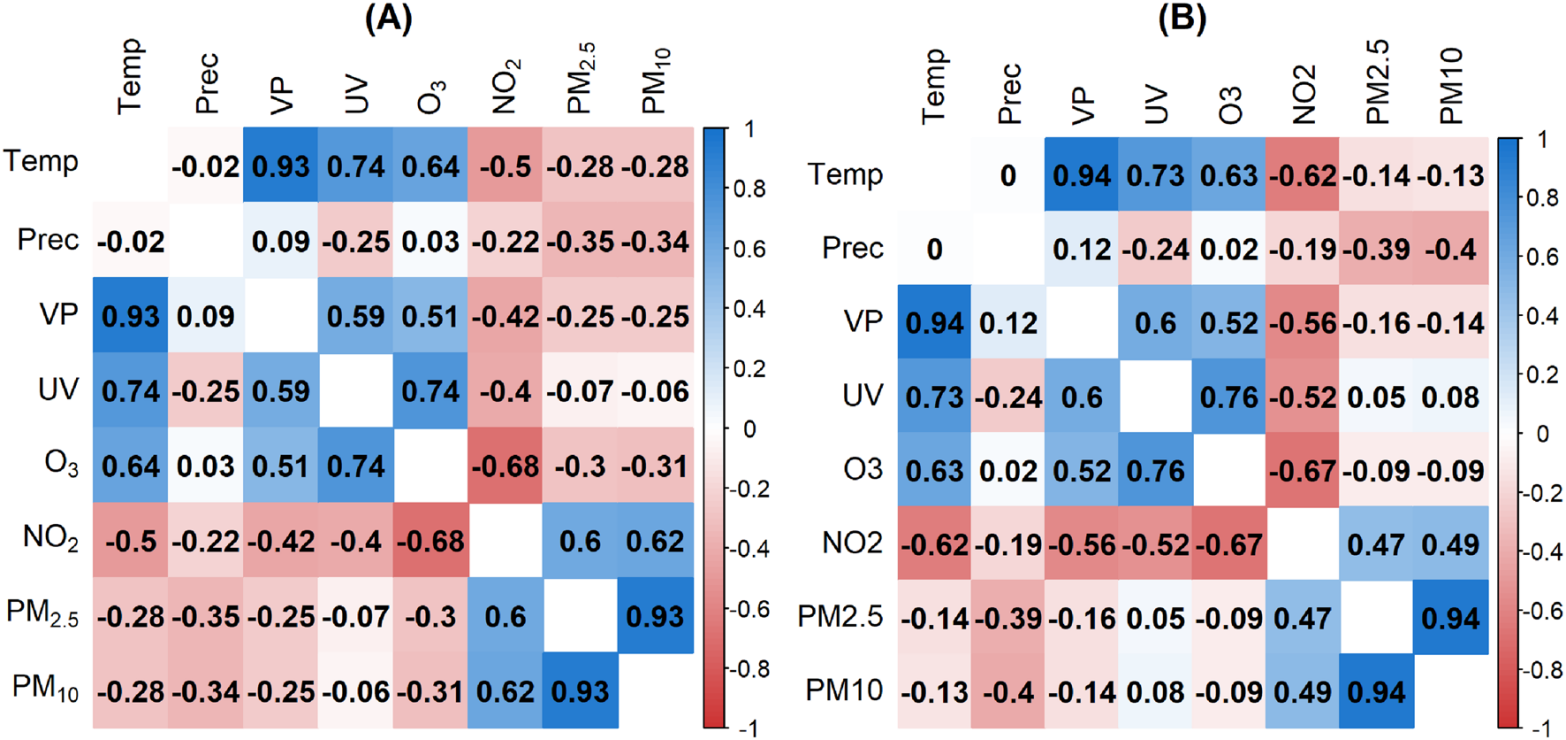
Pearson correlation matrices based on daily measurements aggregated by postal code areas in hot (**A**) and cold (**B**) spots as obtained from the LISA analysis.

Figure 9 displayed the Pearson correlation matrices for the hot and cold spots of the LISA spatial units, similar to the population density categories.

The correlation directions in both matrices were identical. However, the correlation coefficients for $\text{NO}_2$ with *Temp*, *VP*, and *UV* were higher for the cold spots. In addition, the $PM_{2.5}$ and $PM_{10}$ correlation values with all other stressors were overall slightly higher in the hot spots than in the cold spots.

We also generated cross-correlation plots for $\text{NO}_2$ and $\text{O}_3$ in 2018, categorized by both population density and hot and cold spots. However, the cross-correlations do not showed strong changes in the associations between environmental stressors.

## Discussion

**Figure 10:**
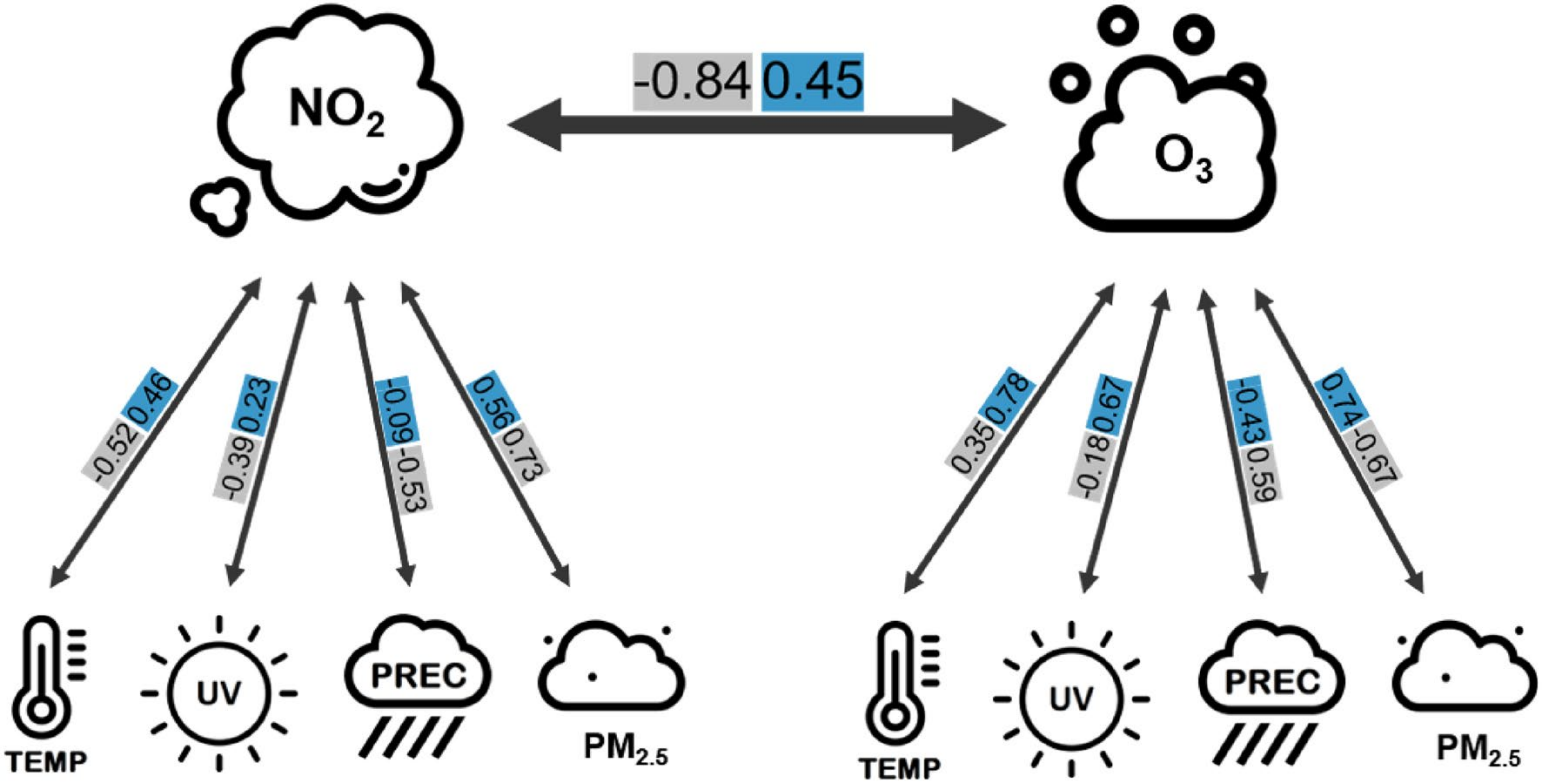
Illustration of the correlations between $\text{NO}_2$ and $\text{O}_3$ in January (gray) and July (blue). The correlation values originated from Fig. 3. This diagram had been designed using images from flaticon.com.

Meteorological and air pollution variables were strongly correlated between and among themselves, with specific seasonal and spatial features. For example, $\text{NO}_2$ and $\text{O}_3$ were strongly interdependent, and the Pearson correlation varied with time. In January, there was a negative correlation of −0.84, whereas in July, the correlation coefficient was 0.45. Figure 10 illustrated that $\text{NO}_2$ and $\text{O}_3$ correlated not only with each other but also with other environmental stressors. It is particularly intriguing to note the contrasting values of the two months, as their correlation directions often differed.

The cross-correlation showed a noticeable change in the correlation direction for $\text{O}_3$ and $\text{NO}_2$. Spatially, $\text{NO}_2$, $PM_{2.5}$, and $PM_{10}$ concentrations were significantly higher in urban than in rural regions. For $\text{O}_3$, the effect is reversed. This result is also confirmed by LISA analysis, where distinct hot and cold spots of the different environmental stressors could be identified. In addition, the Pearson correlation coefficients suggest that $PM_{10}$ variation was almost entirely explained by $PM_{2.5}$ and vapor pressure by temperature.

It is essential to emphasize that correlation does not necessarily imply causality. Correlation means that a change in one variable is related to another variable. However, correlation does not mean that one variable directly influences the other. Thus, there is not necessarily a cause-and-effect relationship. A third variable could affect the two variables that are supposed to be causally related.

Some atmospheric processes can explain the spatio-temporal variability of the correlation coefficients. From an atmospheric chemistry point of view $\text{NO}_2$ and $\text{O}_3$ need to be considered together since they are a function of each other which explains the opposite relationship^51^:

6 $$\begin{aligned} \text{O}_3 + NO \rightarrow {} O_2 + \text{NO}_2. \end{aligned}$$

Their spatio-temporal variability is governed by superimposed emission-based and photochemistry-based regimes^52^. The predominantly anthropogenic nitrogen oxide ($NO_x$) emissions and the resulting $\text{NO}_2$ concentrations have a pronounced seasonal cycle, with higher values in winter than in summer^53^. This is due to the fact that in addition to the higher emissions also the lifetime of $\text{NO}_2$ is longer in winter (about 21 h) than in summer (about 6 h)^54,55^. Peak $\text{NO}_2$ concentrations in winter are resulting from superimposed atmospheric inversion conditions. The photochemically produced tropospheric $\text{O}_3$ exhibits higher levels in the summer months when there is more solar radiation. Nevertheless, the production depends strongly and non-linearly on precursors like $NO_x$ and volatile organic compound (*VOC*) concentrations as well as meteorological conditions. However, photolysis of $\text{NO}_2$ is the primary chemical source of tropospheric ozone^51,56^.

Consequently, the relationship between *O*3 and $\text{NO}_2$ is complex, influenced by a variety of factors and thus needs to be distinguished between rural and urban areas. Since $NO_x$ are emitted from traffic, industrial processes, and other human activities the resulting $\text{NO}_2$ concentration are higher in urban areas and industrial agglomerations as can be inferred from Fig. 7. This is in line with the findings of^23^ where the urban pollution island of the Stuttgart city region could be delineated from satellite.

Ozone in urban areas is primarily formed as a secondary pollutant through chemical reactions involving precursor pollutants, especially during sunny, warm weather conditions and can be transported to rural areas, affecting rural air quality. Paradoxically, locally high emissions of $NO_x$, such as from traffic, tend to favor ozone destruction in urban areas, resulting in $\text{NO}_2$ formation. As can be seen in Fig. 7 this results in ozone cold spots in urban areas. In rural areas, natural sources like vegetation (emitting biogenic volatile organic compounds) and soil contribute to ozone formation. $\text{O}_3$ levels tend to be higher in rural areas where there are fewer local emissions of *NOx* to destroy any $\text{O}_3$ that was photochemically produced. As can be inferred from Fig. 7 the Black Forest mountain range depicts an $\text{O}_3$ hot spot in BW due to the high solar irradiation and the abundance of biogenic volatile organic substances *BVOC* ozone precursors. This is also substantiated in Table 2: the more inhabitants there are, the less $\text{O}_3$ and the more $\text{NO}_2$ occur.

The anticorrelation of $\text{O}_3$ and $\text{NO}_2$, for the reasons outlined above, can be confirmed for all temporal and spatial aggregation levels in the study: Fig. 2 (entire BW, entire period), Fig. 3 (entire BW; Jan, Apr, Oct), Fig. 8 (population density classes, entire period) and Fig. 9 (hot and cold spots; entire period) except for Fig. 3 (entire BW, Jul) where a positive correlation is reported. The negative correlations agree with in-situ measurements of $\text{O}_3$ and $\text{NO}_2$ for Munich, a similar city region to Stuttgart at almost the same latitude, with −0.58 and −0.64 for the period January to July 2019 and 2020, respectively^57^. The same authors find a positive correlation of $\text{O}_3$ and *Temp* of 0.67 and 0.49 for the time periods given and addresses also the interannual variability. This agrees with r = 0.62 in our study for the period 2010 to 2018. Further process-oriented regimes can be identified in the results of the study. Temperature-dominated effects can best be seen in the Pearson correlation matrix for July (Fig. 3C). In the first column the variables *VP*, *UV*, $\text{O}_3$, $\text{NO}_2$, $PM_{2.5}$ and $PM_{10}$ are all positively correlated with *Temp*, partly because they are produced by photochemical processes and linked high solar irradiance ($\text{O}_3$, $\text{NO}_2$, *UV*) or increased by dry weather conditions ($PM_{2.5}$ and $PM_{10}$)^1^. In the second column the effect of wet deposition and cleaning effect is evident by the negative correlation of all air pollutants with precipitation. *UV* shows the strongest negative correlation, due to the presence of clouds. Emission-dominated variables and effects can best be seen in winter (Fig. 3A). It can be inferred that low temperatures, low precipitation and low water pressure favor high concentrations of $\text{NO}_2$, $PM_{2.5}$ and $PM_{10}$, partly due to increased heating, longer photochemical lifetimes and accumulation under inversion conditions or low windspeeds^1,55^. More previous studies have discussed the effect of meteorological conditions on the concentration of atmospheric pollutants and meteorological variables such as wind direction, wind speed and precipitation that have a constraining effect on atmospheric pollutant concentrations, but not a simple linear relationship^20,21,58^. This study is limited to daily data. For further studies, hourly observations could be considered as applied in^10^. The different stressors usually interact differently during the day and at night since e.g. anthropogenic emissions exhibit a pronounced daily cycle and photochemical reactions are confined to sunlit conditions^26^. Another potential extension of the current analysis is to expand the study area to encompass all of Europe. The study uses air pollution and meteorological data that represents background conditions and mesoscale variability. As such air pollution from point sources or along roads cannot be resolved. However, such data is not yet available to our knowledge for the entire BW and the time period under investigation. A possible addition to the variables considered could be wind speed, wind direction and boundary layer height since these parameters have shown a large impact on the variability of particulate matter^59,60^. Furthermore, boundary layer height and $\text{O}_3$ showed the strongest positive correlation among all the analyzed variables in^57^. The Pearson correlation assumes a linear relationship between two continuous variables. Linearity was deemed sufficient for our initial analysis of the internal dependencies of environmental stressors, although there are other correlation coefficients like Spearman correlation that deal with nonlinear associations. We recommend using nonlinear statistical methods such as generalized additive models with splines for advanced studies of air pollution and health factors.

## Conclusion

Selecting the appropriate variables for a statistical model can be a challenging task. This paper offers decision-making assistance for upcoming analyses describing the health effects of environmental stressors. Including a single environmental variable in the model may result in information loss, while including too many variables may lead to correlations and biases. Finding the right balance is important. The optimal choice of variables relies on the specific research question and the given data. However, this paper provides recommendations regarding the variable selection that can be considered. In this work, it turned out to be sufficient to consider $PM_{2.5}$. $PM_{10}$ has larger particles but almost identical temporal and spatial characteristics. The only possible deviation would be for Saharan dust^61,62^. The variables *VP* and *Temp* show strong similarities by design so that future investigations can be limited to the temperature.

The opposite relationship between $\text{NO}_2$ and $\text{O}_3$ was confirmed both temporally and spatially. $\text{NO}_2$ is more often observed in metropolitan areas and $\text{O}_3$ in rural areas. How can this knowledge be addressed in a future model describing the health effects of environmental stressors? For future analyses, we propose incorporating interaction terms to effectively illustrate the relationships between the two variables, $\text{NO}_2$ and $\text{O}_3$, and their impact on the dependent variable. Considering only one environmental stressor in a future model may lead to loss of information and confusion in interpreting. Moreover, including both variables in the model without an interaction term is not advisable, as high Pearson correlation coefficients may cause bias. Based on this, we recommend using an interaction term between $\text{NO}_2$ and $\text{O}_3$.

It is important to note that different stressors have different health effects. Although the previous analysis suggests that $\text{NO}_2$ and $\text{O}_3$ are opposite, they have different impacts on human health^40^. This fact makes the choice of model and the interpretation of the relationships more complex and must be considered in future analyses.

When applying the LISA model, we found substantial spatial differences in some variables (e.g. $PM_{2.5}$), but not in others (*UV*) between urban and rural areas. This indicates that this spatial variation can be statistically exploited for epidemiological studies. Notably, a large fraction of postal code regions show lack of coherence with their neighbors, as can seen from the high proportion of uncolored areas in Fig. 7. In addition, we also identified clear patterns of LISA hot and cold spots, particularly in urban areas, mountainous regions Schwarzwald, and Schwäbische Alb. All of these identified patterns show positive autocorrelations, and no negative autocorrelation was observed.

We spatially categorized the state of Baden-Wüttemberg in two ways: first, by population density (Fig. 2 and table 2) and second, by LISA hot and cold spots (Fig. 7 and table 3). The two categorizations matched spatially well for some air pollution variables (e.g. $PM_{2.5}$ and $\text{NO}_2$) and less well for some meteorological variables (e.g. *UV*).

To conclude, it will be straightforward to implement the principle findings of our study, namely (a) the temporal coherence of stressor patterns, and (b) the spatial clustering into statistical models for the epidemiological study of stressor effects on human health, e.g. by affording the necessary spatial categorical variables and the opportune interaction terms into the statistical models.

### Supplementary Information

Supplementary Information.

## Data Availability

The data that support the findings of this study are available on request from the corresponding author LH.

## Abbreviations

BVOC: Biogenic volatile organic substances
BW: Baden-Württemberg
CAMS: Copernicus Atmosphere Monitoring Service
CCF: Cross-correlation function
COVID-19: Coronavirus SARS-CoV-2
$$\text{CO}_2$$: Carbon dioxide
C3S: Copernicus Climate Change Service
DLR: German Aerospace Center
ECMWF: European Centre for Medium-Range Weather Forecasts
EEA: European Environment Agency
LISA: Local indication of spatial association
$$\text{NO}_2$$: Nitrogen dioxide
$$\text{NO}_x$$: Nitrogen oxide
$$\text{O}_3$$: Ozone
PCA: Principal component analysis
$PM_{2.5}$: Particulate matter with a diameter of 2.5 micrometers or smaller
$PM_{10}$: Particulate matter with a diameter of 10 micrometers or smaller
Prec: Precipitation
sd: Standard deviation
r: Correlation coefficient
Temp: Temperature
UV: ultraviolet radiation
VOC: Volatile organic compound
VP: Vapor pressure
WHO: World Health Organization
