# Supplementary material for: Investigating the spatiotemporal associations between meteorological conditions and air pollution in the federal state Baden-Württemberg (Germany)
Source: Sci Rep. 2024 Mar 12;14:5997. doi: 10.1038/s41598-024-56513-4 (PMC10933279; doi:10.1038/s41598-024-56513-4)
Supplement: Supplementary file 1 — Supplementary Information. [file 41598_2024_56513_MOESM1_ESM.pdf]

Investigating the spatiotemporal associations between meteorological conditions and air pollution in the federal state Baden-Württemberg (Germany)

Leona Hoffmann[1*], Lorenza Gilardi[2], Marie-Therese Schmitz[3], Thilo Erbertseder[2], Michael Bittner[2], Sabine Wüst[2], Matthias Schmid[3], Jörn Rittweger[1,4]

[1]Institute of Aerospace Medicine, German Aerospace Center (DLR), Cologne, Germany.
[2]German Remote Sensing Data Center, German Aerospace Center (DLR), Weßling, Germany.
[3]Institute of Medical Biometry, Informatics and Epidemiology, University Hospital Bonn, Bonn, Germany.
[4]Department of Pediatrics and Adolescent Medicine, University Hospital Cologne, Cologne, Germany.

*Corresponding author. E-mail: leona.hoffmann@dlr.de;

Supplementary Information

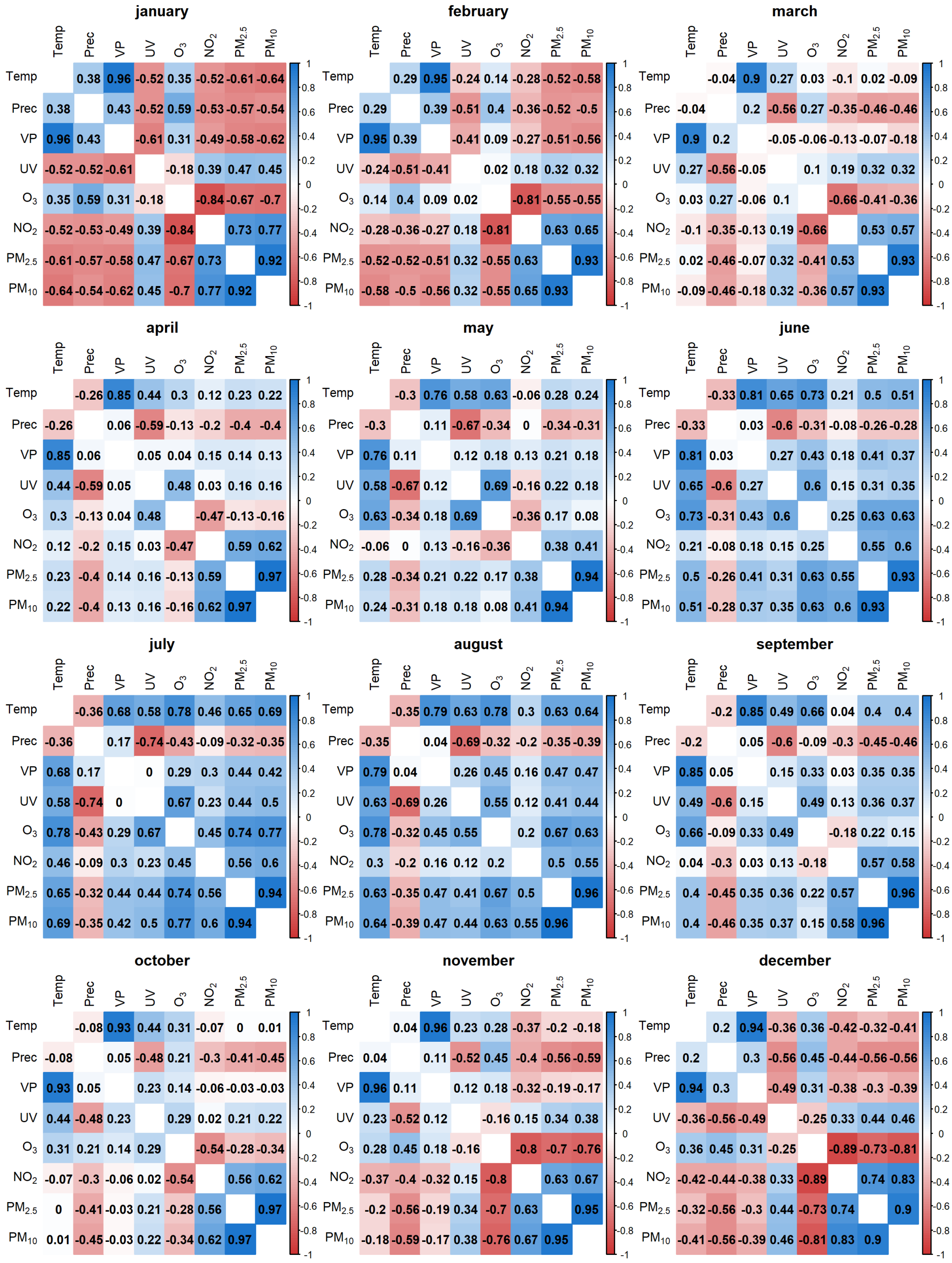

Fig. S1 Pearson correlation matrices based on daily measurements from 2010 to 2018 across BW split by all months.
